# Is preexisting mental illness associated with lower patient satisfaction for older trauma patients? A cross-sectional descriptive study

**DOI:** 10.1186/s12888-021-03071-y

**Published:** 2021-01-30

**Authors:** Constance McGraw, Jennifer Pekarek, Diane Redmond, Rebecca Vogel, Allen Tanner, David Bar-Or

**Affiliations:** 1 Trauma Research Department, St. Anthony Hospital, CO Lakewood, USA; 2 Trauma Research Department, Penrose-St. Francis Health Services, Colorado Springs, CO USA; 3 Trauma Services Department, St. Anthony Hospital, Lakewood, CO USA; 4 Trauma Services Department, Penrose St.-Francis Health Services, Colorado Springs, CO USA

**Keywords:** Functional status, Depression, Older adults, Activities of daily living

## Abstract

**Background:**

The purpose of this study was to examine if satisfaction with care differs among older trauma patients with and without preexisting mental illness (PMI+/PMI-).

**Methods:**

Data from two level I trauma centers were examined from 11/2016 through 12/2017. Trauma patients ≥55 years were included and satisfaction of those who had a diagnosis of mental illness prior to the trauma admission (PMI+) to those without a diagnosis (PMI-) (*n* = 299; 62 PMI+ and 237 PMI-) were compared. Enrolled patients completed the Family Satisfaction with Advanced Care Cancer Scale Patient Survey (FAMCARE-P13) prior to discharge. Associations between mental illness status and patient baseline characteristics, overall mean satisfaction, and mean satisfaction by question were compared. Generalized linear models adjusted for differences in patient satisfaction by mental illness status. Analyses were stratified by hospital to account for the interaction between hospital and mental illness status.

**Results:**

Compared to PMI- patients, PMI+ patients were more likely to be younger, female, have multiple comorbidities, and to report lower overall satisfaction with care. Among PMI+ patients, the most common diagnoses were depression and anxiety. After adjustment, PMI+ was associated with lower patient satisfaction at hospital 1; after examining individual questions lower satisfaction was associated with information provided on procedures and questions surrounding “Physical care.” Conversely, PMI+ did not affect satisfaction at hospital 2 after adjustment.

**Conclusions:**

At hospital 1, room for improvement was identified in providing information about prognosis and procedures, symptom management, and continuity of care. Reexamining practices for older PMI+ trauma patients is warranted.

**Supplementary Information:**

The online version contains supplementary material available at 10.1186/s12888-021-03071-y.

## Background

The growing number of injured patients admitted with preexisting mental illness (PMI) has become a concern for trauma centers across the United States in recent years [1]. According to an estimation from the National Trauma Data Bank in 2016, 10% of patient records contained a comorbidity related to a major mental illness and estimates in the general population have reported between 18 and 26% [2–4]. PMI can cause additional burdens for hospitalized trauma patients and has been associated with increased hospital length of stay (LOS), complications, and higher costs of care, compared to those without mental illness [5–7]. These challenges are amplified for older trauma patients, who often already face functional and cognitive decline. The Centers for Disease Control and Prevention has reported that 20% of people aged ≥55 years old suffer from mental illness, yet there is a paucity of research on PMI in older (trauma) patients [8].

Furthermore, studies show that a diagnosis of depression increases the risk of a fall or other traumatic injury particularly for older adults, as well as causing delays in functional recovery, an increased risk of a repeat fall during hospitalization, and distorted perceptions of care [9–14]. Although mental health examinations in-house are paramount for injury prevention in the elderly, studies suggest that PMI in elderly patients is frequently unaddressed by the trauma surgical staff [9, 15]. Similar findings have been echoed in a systematic review by Mitchell and colleagues, where across 31 studies, frequent disparities existed in the physical healthcare provided to patients with mental illness compared to those without one [16]. Further complicating care of patients with PMI, older adults are also less likely than their younger counterparts to seek out help for managing their mental illness [9], potentially leading to missed opportunities for appropriate management or skewed perceptions of hospital care. Accordingly, this study explored the experience of older trauma patients with PMI while hospitalized.

The objectives of the study included: 1) measure satisfaction of older adult patients with preexisting mental illness compared to patients without mental illness; 2) characterize patients with preexisting mental illness; 3) identify areas for improvement in patient satisfaction.

## Methods

### Study design

This prospective cross-sectional study included trauma patients aged ≥55 years, admitted between November 1, 2016-December 31, 2017, across two level I trauma centers. Included patients were then categorized into either PMI+ (patients who had ≥1 diagnosis of mental illness prior to the trauma admission) or PMI- (patients without a diagnosis) based on information contained in their medical records. Further details on the inclusion and exclusion criterion, and the processes and details for administering the surveys (including their theoretical framework and initial findings) are outlined in a previous study [17]. Briefly, eligible patients (trauma admissions ≥55 years old) were approached by the study coordinators at each hospital to be enrolled. If the patient was unable to consent, they could be enrolled by proxy. Patients with severe cognitive impairment as well as those who did not meet state trauma registry inclusion criteria were excluded. Consented patients were administered the FAMCARE-P13 (Family Satisfaction with Advanced Cancer Scale, Patient), prior to discharge. The FAMCARE-P13 survey measures the degree of patient, satisfaction with information provided, availability of hospital staff, physical care management, and psychosocial care and has a possible score range of 1–65 (13 questions), with higher scores reflecting higher levels of satisfaction [18–20]. The survey questions also can be grouped into the following conceptual structures: Information giving, Availability of care, Physical care, and Psychosocial care [21].

These analyses were conducted in the context of a larger prospective study. Because this was an analysis from an ongoing study, a formal sample size calculation was not performed. Patients were provided written informed consent prior to being enrolled and surveyed. This study was carried out according to the STROBE-guidelines on the reporting of observational studies and was approved by institutional review boards at the participating centers.

### Standard of care

According to the American College of Surgeons’ (ACS) Guidelines for level I trauma centers, hospitals are required to screen at least 80% of all trauma admissions using screening, brief intervention, and referral to treatment (SBIRT), to identify those with problematic mental illnesses [22]. The exact process for the screening and evaluation for mental illness is left up to each trauma center. Across the hospitals in this study, a team of social workers and a clinician are usually responsible with screening patients for alcohol, substance use, and/or mental health concerns, and discerning the need for a brief or formal intervention depending on the severity of the concern.

### Covariates and outcomes

The following covariates were collected on each patient from the trauma registry: sex, age (55–65, > 65), race, injury severity score (ISS, 1–15, ≥16), hospital length of stay (LOS), ICU LOS, cause of injury (fall vs. high acuity vs. sport vs. other), hospital discharge destination (home/health vs. skilled nursing facility vs. long term care vs. hospice), the presence of the following comorbid conditions: a history of mental illness, smoking, dementia, diabetes, chronic obstructive pulmonary disease (COPD), hypertension, functionally dependent health status, anticoagulant use, congestive heart failure (CHF), alcoholism, and an advanced directive limiting care.

Covariates collected from patient EMRs included: specific mental illness diagnosis, medical insurance, mental illness management (behavioral health assessment, psychiatric consultation, discharge plans), mental illness medication, and the history of the diagnosis (longstanding mental illness: ≥2 years or newer diagnosis < 2 years), traumatic brain injury (TBI) diagnosis.

The primary outcome variable was overall mean (SD) satisfaction score for patients from the FAMCARE-P13 survey and the secondary outcome was patient satisfaction by survey question.

### Statistical analyses

Satisfaction and in-hospital patient characteristics were compared univariately by the patient’s PMI status. Descriptive statistics were analyzed using Chi-squared tests or Fisher’s exact tests for categorical variables. Student’s t-tests, one-way ANOVAs, Wilcoxon two-sample tests, or Kruskal Wallis tests were used for continuous variables, as appropriate. Data are displayed as means and standard deviations (SD), medians and interquartile ranges (IQR), or proportions.

Generalized linear models were used to determine whether differences in patient satisfaction existed between the PMI+ and PMI- patients. Entry and exit criterion were set to *p* = 0.2 and *p* = 0.1, respectively, and manually entered into the model. Any significant interactions between PMI status and other covariates on patient satisfaction were explored. PMI status was the primary exposure variable and thus was automatically included in each model. SAS 9.4 (Cary, NC) was used for all analyses. Two-tailed tests with an alpha < 0.05 were used for all tests.

## Results

Of the 309 patients surveyed during the study period, 10 were excluded; 9 patients upon final review did not meet state trauma registry inclusion criteria; 1 patient did not have enough information listed in the EMR to make an informed decision on mental illness diagnosis. After exclusions, there were 299 patients included in the study and of them, 62 (21%) were PMI+ and 237 (79%) were PMI-. Overall, the majority of patients were female (55%), were a mean (SD) age of 73.5 (10.9) years, had a median (IQR) ISS of 9 (5–10), spent a median of 4 (3–6) days hospitalized, and had a mean satisfaction of 82.0% (14.2).

### Patient characteristics by PMI status

Table 1 reports any differences in characteristics between patients by PMI status. Compared to patients who were PMI-, PMI+ patients had significantly lower mean (SD) satisfaction (82.9% (13.5) vs. 78.7% (16.2), *p* = 0.04), were more likely to be in the age range of 55–64 vs. ≥65 years (21% vs. 35%, *p* = 0.02), were significantly more likely to be female (49% vs. 74%, *p* = 0.003), to have ≥2 comorbidities (36% vs. 92%, *p* < 0.001), and to have private insurance (30% vs. 45%, *p* = 0.03). There were no other significant differences in injury characteristics or outcomes found between groups.

**Table 1:** Patient Characteristics by Preexisting Mental Illness Status

Characteristics, n (%)	PMI+ *N* = 62 (21%)	PMI- *N* = 237 (79%)	*P*-value
Overall, mean (SD) patient satisfaction	78.6% (16.3)	82.9% (13.5)	**0.03**
Sex (female)	46 (74%)	117 (15%)	**0.003**
Age			**0.02**
*55–64*	22 (35%)	49 (21%)	
*≥ 65*	41 (65%)	188 (79%)	
Injury severity score			0.97
*1 to 15*	56 (89%)	211 (89%)	
*≥ 16*	7 (11%)	26 (11%)	
Mechanism of injury (Fall)	50 (79%)	168 (71%)	0.57
LOS	5 (3–7)	4 (3–6)	0.35
ICU LOS	3 (2–3)	2 (2–3)	0.30
ICU stay, n (%)	17 (27%)	89 (38%)	0.12
Hospital, n (%)			**0.04**
*Hospital 1*	31 (50%)	85 (64%)	
*Hospital 2*	31 (50%)	152 (36%)	
Comorbidity count			**< 0.001**
*≤ 1*	5 (8%)	151 (64%)	
*≥ 2*	57 (92%)	86 (36%)	
Comorbidities (yes vs. no)			
*Smoker*	6 (10%)	20 (8%)	0.74
*Hypertension*	33 (54%)	124 (53%)	0.82
*Dementia*	5 (8%)	7 (3%)	0.07
*Diabetes*	15 (24%)	35 (15%)	0.07
*COPD*	9 (15%)	18 (7%)	0.08
*CHF*	5 (8%)	7 (3%)	0.08
Type of insurance			0.11
*Medicaid*	2 (3%)	11 (5%)	> 0.99
*Medicare*	29 (47%)	143 (60%)	0.05
*Medicare combination*	18 (29%)	87 (37%)	0.26
*Private*	28 (45%)	72 (30%)	**0.03**
*Uninsured*	0 (0%)	6 (3%)	0.35
*Other*	3 (5%)	5 (2%)	0.37
TBI diagnosis	11 (18%)	55 (23%)	0.34
Discharge location			0.33
*Home/home health*	24 (38%)	111 (47%)	
*LTC/SNF*	38 (60%)	125 (53%)	
*Rehab*	12 (19%)	33 (14%)	
*Hospice*	1 (2%)	1 (0.4%)	

*PMI* Preexisting mental illness status (+ yes; −no), *SD* Standard deviation, *LOS* Hospital length of stay, *ICU* Intensive care unit, *COPD* Chronic obstructive pulmonary disease, *CHF* Congestive heart failure, *TBI* Traumatic brain injury, *LTC* Long-term care, *SNF* Skilled nursing facility

### Characteristics of PMI+ patients

The most common mental illness diagnosis reported among PMI+ patients was depression (70%), followed by anxiety (35%), and then dementia (8%), and in most (89%) patients, the diagnosis was longstanding (≥2 years) (Table 2). Fourteen (23%) PMI+ patients received a behavioral health assessment (hospital 1, *n* = 2 and hospital 2, *n* = 12); the most common trigger for the assessment was “Coping concerns with acute change in activities of daily living” (62%), while the remaining triggers included: “Safety concerns at home” (15%); “Evaluation outbursts in hospital”(8%); “Long history of depressive symptoms” (8%), and “Suicide attempt” (8%).

**Table 2:** Characteristics of Trauma Patients with ≥1 Preexisting Mental Illness

Characteristics	*N* = 62	Percent
Number of diagnoses		
1	40	65%
*2*	20	32%
*3*	2	3%
Types of Diagnoses^a^		
*Depression*	45	73%
*Anxiety*	22	35%
*PTSD*	2	3%
*Dementia*	4	6%
*Bipolar disorder*	2	3%
*Chronic pain disorder*	2	3%
*Fibromyalgia*	3	5%
*Dissociative disorder*	1	2%
*Personality disorder*	1	2%
*Drug abuse*	1	2%
*Mood disorder*	1	2%
Management		
*Psychiatric consultation*	5	8%
*Behavioral health assessment*	14	23%
*Discharge plan*	39	63%
PMI was long-standing (≥2 years)	55	89%
PMI was being managed with medication	48	77%

*PTSD* Post-traumatic stress disorder, *PMI* Preexisting mental illness status (+ yes; −no). ^a^Patients can have more than one diagnosis

### Adjusted modeling for satisfaction

Satisfaction by PMI status was modified by hospital; therefore, each adjusted model was also hospital-stratified, and data are reported in Table 3 (hospital 1) and 4 (hospital 2). After adjustment for smoking status (yes vs. no), PMI+ patients at hospital 1 had significantly lower overall satisfaction, than PMI- patients, respectively (69.8% vs. 79.5%, *p* < 0.003, Table 3). Conversely at hospital 2, after adjustment for smoking status and hospital LOS, PMI+ patients did not have significantly lower satisfaction than PMI- patients (73.5% vs. 74.7%, *p* = 0.61, Table 4). Please refer to Additional file 1 for supplementary 1 table on differences in patient characteristics by hospital.

**Table 3:** Adjusted Model Examining Predictors of Satisfaction: Stratified by Hospital 1

Exposure variable & covariates	Mean Patient Satisfaction% (LSM: (95% CI))	*P*-value
PMI+ vs. PMI-	69.8 (62.1–77.4) vs. 79.5 (73.7–85.2)	**0.003**
History of smoking (yes vs. no)	69.9 (60.1–80.0) vs. 84.5 (81.3–87.7)	**0.004**

*LSM* Least squares mean, *CI* Confidence interval, *PMI* Preexisting mental illness status (+ yes; −no)

**Table 4:** Adjusted Model Examining Predictors of Satisfaction: Stratified by Hospital 2

Exposure variable & covariates	Mean Patient Satisfaction% (LSM: (95% CI))	*P*-value
PMI+ vs. PMI-	73.5 (68.7–78.3) vs. 74.7 (71.4–78.0)	0.61
History of smoking (yes vs. no)	68.5 (62.5–74.5) vs. 79.7 (77.2–82.2)	**0.005**
Hospital LOS (≤4 days vs. > 4 days)	75.5 (71.8–79.1) vs. 72.8 (68.8–76.8)	0.14

*LSM* Least squares mean, *CI* Confidence interval, *PMI* Preexisting mental illness status (+ yes; −no), *LOS* Length of stay

### Satisfaction by survey question and hospital

Table 5 outlines satisfaction by survey question between PMI+ and PMI- patients at hospital 1. Under Information giving, PMI+ patients had significantly lower mean satisfaction than PMI- patients with question 4, “Information provided about your prognosis (75.7% vs. 85.6, *p* = 0.03, Table 5) and question 6, “Information given about your procedures” (73.3% vs. 88.4%, *p* = 0.002). Interestingly, PMI+ patients also had significantly lower satisfaction than PMI- patients for all questions (1, 5, 7, 10, 11) related to Physical care. Please refer to Additional file 1 (supplementary table 2) for patient satisfaction by survey question at hospital 2.

**Table 5:** Satisfaction by Survey Question at Hospital 1

Conceptual structure, Item number	PMI+, *N* = 31 (27%) Mean% (SD) Satisfaction	PMI-, *N* = 85 (73%) Mean% (SD) Satisfaction	*P*-value
Overall satisfaction at Hospital 1	78.9 (19.6)	88.0 (13.7)	**0.006**
**Information giving**			
2 Information given about how to manage pain	82.5 (23.8)	87.5 (17.6)	0.23
4 Information provided about your prognosis	75.7 (24.5)	85.6 (18.3)	**0.03**
6 Information given about your procedures	73.3 (32.1)	88.4 (18.1)	**0.002**
12 Information given about side effects	78.3 (19.4)	83.3 (20.0)	0.24
9 Answers from health professionals	86.7 (19.4)	88.7 (18.0)	0.60
**Availability of care**			
3 The availability of nurses to answer your questions	85.5 (22.2)	91.5 (15.7)	0.11
8 The availability of doctors to answer your questions	77.5 (27.4)	83.3 (19.9)	0.22
**Physical care**			
1 How thoroughly the doctor assessed your symptoms	82.3 (28.3)	92.9 (17.6)	**0.02**
5 Speed with which symptoms were treated	75.8 (25.0)	88.4 (15.8)	**0.002**
7 The way procedures were performed	80.8 (21.5)	89.5 (18.4)	**0.04**
10 Referrals to specialists	77.0 (28.8)	90.1 (5.5)	**0.009**
11 The way tests and treatments are followed-up by the doctor	75.8 (28.2)	86.3 (18.2)	**0.02**
**Psychosocial care**			
13 The way the family was included in treatment and care decisions	83.0 (25.5)	89.8 (18.3)	0.14

*PMI* Preexisting illness status (+ yes; −no), *SD* Standard deviation

## Discussion

Despite evidence showing that patients with preexisting mental illness tend to have worse outcomes and a more challenging hospitalization [5, 7], there is a scarcity of research on their perception of the care experience. To our knowledge, this is one of the first studies to examine satisfaction and characteristics of older trauma patients with PMI. Across two Level I trauma centers, of those who were PMI+, the most common diagnosis was depression and patients tended to be females who were between 55 and 64 years old, and had multiple comorbidities compared to those who were PMI-. Only a small fraction of PMI+ patients qualified for a behavioral health assessment; this was particularly true at hospital 1, where only two PMI+ patients had an assessment, and reported significantly lower satisfaction with both delivery of information and physical care compared to PMI- patients.

Several of these patient characteristics have been substantiated in the published literature [5–7, 23–25]. Townsend and colleagues (2017) reported on characteristics of PMI+ trauma patients using the Nationwide Inpatient Sample (2012) and found that 44% of trauma patients ≥18 years of age were PMI+ across 36.5 million patients, and similarly, PMI+ trauma patients were significantly more likely to be female, to be an average age of 61 years (vs. 56 years), and have a higher number of comorbidities compared to PMI- trauma patients [5]. In a single center study, Weinberg et al. (2016) described the prevalence and characteristics of psychiatric illness among orthopedic polytrauma patients and also discovered a significantly higher percentage of PMI+ patients (vs. PMI-) were female (38.5% vs. 23.7%) and had depression (22.3%) [6].

Interestingly, smoking status was identified as an independent predictor of lower patient satisfaction at both hospitals. In this study, patients with a history of smoking had significantly more comorbidities compared to non-smokers, and significantly more also had a history of alcoholism, potentially contributing to lower satisfaction. Life satisfaction in older populations has been linked to activities that both temporarily alleviate and contribute to stress and pain, including smoking and alcohol consumption [26]. Thus, a history of smoking has been associated with lower life satisfaction [26], as well as lower patient satisfaction [17].

Nonetheless, it remains essential for trauma centers to target unmet patient needs, in order to tailor care and improve satisfaction. The identification of the most common trigger for behavioral health assessments, “Coping concerns with acute changes in ADLs”, should serve as a starting point for both hospitals to best serve their older PMI+ patient population. Because this was the most common trigger, it can be surmised that these concerns are insidious among the majority of older patients, both PMI+ and PMI-. A decline in ADLs for older adults, but particularly those with dementia, has been shown to negatively affect their quality of life and perception of care [27] and furthermore, a diagnosis of depression or anxiety is associated with an increased risk of cognitive and physical decline over time for elderly patients [25, 28–33]. In this study, over half of PMI+ patients had a diagnosis of depression. Depressed patients more frequently perceive events in their life as negative compared to those without depression [34, 35] and those with depressive symptoms have previously been found to be less satisfied with communication from their provider [36].

There is room for improvement across both hospitals. At hospital 2, although no difference in patient satisfaction across groups (PMI+ vs. PMI-) was identified, overall satisfaction was significantly lower than at hospital 1. This finding may be due to several reasons, one being the older patient population at hospital 2; older patients generally have less mobility, translating to lower overall life and thus patient satisfaction [26, 27, 32]. Second, patients at hospital 2 also tended to be managed on the floor, whereas at hospital 1, which included a younger population, patients had a slightly higher injury severity and typically went to critical care. At hospitals 1 and 2, satisfaction with communication of information on prognosis was significantly lower, which was identified as an area of lower patient satisfaction in a recent study on trauma patients [17], and in other patient populations [36–38]. Although these patients could in fact have been provided relevant information during their stay, significant complexities still exist in communication of information among PMI+ patients and providers [9, 14–16], which may be more pronounced in the rush of an acute trauma setting.

At trauma centers injuries always take priority and PMI+ patients may not be flagged for a behavioral health assessment, especially if their injury is critical; however, across both trauma centers, assessments only seemed to be triggered if the PMI+ patient was clearly high-risk, potentially leaving many less obvious PMI+ patients with unmet care needs. At hospital 1 specifically, communication and management surrounding pain, prognosis, procedures, and referrals to specialists, contributed to significantly lower satisfaction, suggesting that that providers may not be closing the loop on the continuity of care after managing the injury. Closing the loop should include rigorous screening and assessment, thorough symptom management, as well as appropriate referrals for counseling and other mental health support.

### Limitations

There are a number of limitations in the study. First, the patients responding to the satisfaction surveys may not have been representative of the general population seen across the facilities. Patients who were “very dissatisfied” or “undecided” may have withheld from taking the survey. Second, healthcare surveys administered in person can sometimes artificially inflate the results because patients might believe that their scores will negatively affect the care received; however, by capturing results soon before hospital discharge and without disclosing them to treating staff, a more accurate and unbiased description of patient satisfaction was obtained. Third, because this was a cross-sectional study of two trauma centers, other hospitals with different populations and behavioral health management strategies may not observe the same satisfaction scores; nonetheless, the inclusion of a general, older adult trauma population may help other centers hoping to understand satisfaction and characteristics of trauma patients who are PMI+. Fourth, because data was used from a larger ongoing study, the surveys used were not previously evaluated in a trauma population, or for those who are PMI+ and additional work may be needed to verify the results using an instrument tailored for this population; however, Vogel and colleagues (2019) successfully measured patient and caregiver satisfaction using the FAMCARE surveys in a trauma population [17, 39]. Fifth, because the measurement of mental illness in this population was taken from patients who consented to take a survey, it is likely an underestimation of the true prevalence across these hospitals. Last, SBIRT components were not captured for PMI+ patients and thus compliance with ACS guidelines was not able to be measured.

## Conclusions

Older trauma patients who are PMI+ are frequently overlooked, which can lead to unmet needs and lower satisfaction with care. These findings may be indicative of hospital culture generally, in regard to older patients with preexisting mental illness. Reexamining guidelines and treatment practices for this unique patient population is warranted.

## Supplementary Information

**Additional file 1.**

## Data Availability

De-identified patient data, protocols, and statistical analysis plans are available upon reasonable request by the corresponding author: davidbme49@gmail.com; ORCID ID: 0000–0002-3685-314X.

## Abbreviations

PMI (+/−): Preexisting mental illness diagnosis prior to trauma admission vs. without
LOS: Hospital length of stay
ACS: American College of Surgeons
SBIRT: Screening, brief intervention, and referral to treatment
ICU: Intensive care unit
COPD: Chronic obstructive pulmonary disease
CHF: Congestive heart failure
EMR: Electronic medical record
TBI: Traumatic brain injury
SD: Standard deviation
IQR: Interquartile range
ADLs: Activities of daily living
SNF: Skilled-nursing facility
LTC: Long-term care
PTSD: Post-traumatic stress disorder
LSM: Least squares mean
CI: Confidence interval
