# Supplementary material for: Is preexisting mental illness associated with lower patient satisfaction for older trauma patients? A cross-sectional descriptive study
Source: BMC Psychiatry. 2021 Jan 30;21:67. doi: 10.1186/s12888-021-03071-y (PMC7847564; doi:10.1186/s12888-021-03071-y)
Supplement: Supplementary file 1 — Additional file 1. [file 12888_2021_3071_MOESM1_ESM.docx]

**APPENDIX A: SUPPLEMENTARY FILE # 1**

| Supplementary Table 1. Overall Differences in Patient Characteristics by Hospital | | | |
| --- | --- | --- | --- |
| Patient characteristics, n (%) | Hospital 1, N=116 (39%) | Hospital 2, N=183 (61%) | P-value |
| PMI+ | 31 (27%) | 31 (17%) | **0.04** |
| Overall, mean (SD) patient satisfaction | 85.7 (15.9) | 79.7 (12.5) | **0.003** |
| Sex (female) | 59 (51%) | 103 (56%) | 0.40 |
| Age |  |  | **0.006** |
| *55-64* | 37 (32%) | 33 (18%) |  |
| *≥65* | 79 (68%) | 150 (82%) |  |
| Injury severity score |  |  | 0.11 |
| *1 to 15* | 99 (85%) | 167 (91%) |  |
| *≥16* | 17 (15%) | 16 (9%) |  |
| Mechanism of injury |  |  | **<0.001** |
| *Fall* | 69 (59%) | 149 (81%) |  |
| *High acuity* | 24 (21%) | 23 (13%) |  |
| *Sport* | 23 (20%) | 8 (4%) |  |
| *Other* | 0 (0%) | 3 (2%) |  |
| LOS | 6.2 (3.7) | 4.6 (2.0) | **<0.001** |
| ICU stay, n (%) | 48 (41%) | 56 (31%) | 0.06 |
| Comorbidity count |  |  | 0.28 |
| *≤1* | 56 (48%) | 100 (55%) |  |
| *≥2* | 60 (5%) | 83 (45%) |  |
| Comorbidities |  |  |  |
| *Mental illness* | 31 (27%) | 31 (17%) | **0.04** |
| *Dementia* | 2 (2%) | 10 (5%) | 0.14 |
| *Dependent health* | 3 (3%) | 17 (9%) | **0.03** |
| TBI diagnosis | 29 (26%) | 37 (20%) | 0.27 |
| Discharge location |  |  | **0.002** |
| *Home/home health* | 67 (58%) | 65 (36%) |  |
| *SNF/LTC* | 30 (26%) | 88 (49%) |  |
| *Hospice* | 0 (0%) | 2 (1%) |  |
| *Rehab* | 8 (16%) | 26 (14%) |  |
| Behavioral health assessment | 2 (7%) | 12 (39%) | **0.003** |
| PMI+, preexisting mental illness positive; SD, standard deviation; LOS, hospital length of stay; ICU, intensive care unit; TBI traumatic brain injury; SNF, skilled nursing facility; LTC, long-term care. | | | |

**APPENDIX B: SUPPLEMENTARY FILE # 2**

| Supplementary Table 2. Satisfaction by Survey Question at Hospital 2 | | | |
| --- | --- | --- | --- |
| Conceptual structure, Item number | PMI+, N=31 (17%) Mean% (SD) Satisfaction | PMI-, N=152 (83%) Mean% (SD) Satisfaction | P-value |
| Overall satisfaction | 77.7 (12.3) | 80.1 (12.6) | 0.34 |
| **Information giving** |  |  |  |
| 2 Information given about how to manage pain | 73.4 (18.2) | 77.3 (16.7) | 0.25 |
| 4 Information provided about your prognosis | 72.6 (18.7) | 80.0 (16.2) | **0.02** |
| 6 Information given about your procedures | 79.3 (17.8) | 80.1 (15.5) | 0.81 |
| 12 Information given about side effects | 69.8 (19.3) | 77.2 (17.4) | **0.04** |
| 9 Answers from health professionals | 81.7 (17.3) | 81.0 (15.3) | 0.83 |
| **Availability of care** |  |  |  |
| 3 The availability of nurses to answer your questions | 82.3 (14.7) | 80.9 (13.7) | 0.63 |
| 8 The availability of doctors to answer your questions | 79.5 (19.3) | 82.2 (14.6) | 0.39 |
| **Physical care** |  |  |  |
| 1 How thoroughly the doctor assessed your symptoms | 80.0 (17.9) | 81.0 (13.1) | 0.73 |
| 5 Speed with which symptoms were treated | 75.0 (16.4) | 79.0 (16.7) | 0.24 |
| 7 The way procedures were performed | 76.8 (16.6) | 80.1 (15.0) | 0.29 |
| 10 Referrals to specialists | 75.0 (16.7) | 78.9 (15.3) | 0.23 |
| 11 The way tests and treatments are followed-up by the doctor | 78.7 (15.0) | 90.1 (14.7) | 0.66 |
| **Psychosocial care** |  |  |  |
| 13 The way the family was included in treatment and care decisions | 82.3 (17.3) | 82.9 (13.9) | 0.82 |
| PMI, preexisting illness status (+ yes; -no); SD, standard deviation. | | | |
